# Supplementary figures and images for: Low Adenovirus Vaccine Doses Administered to Skin Using Microneedle Patches Induce Better Functional Antibody Immunogenicity as Compared to Systemic Injection
Source: Vaccines (Basel). 2021 Mar 22;9(3):299. doi: 10.3390/vaccines9030299 (PMC8005075; doi:10.3390/vaccines9030299)

**Figure S1: Graphical representation of immunization regimes and schedules used in this study**

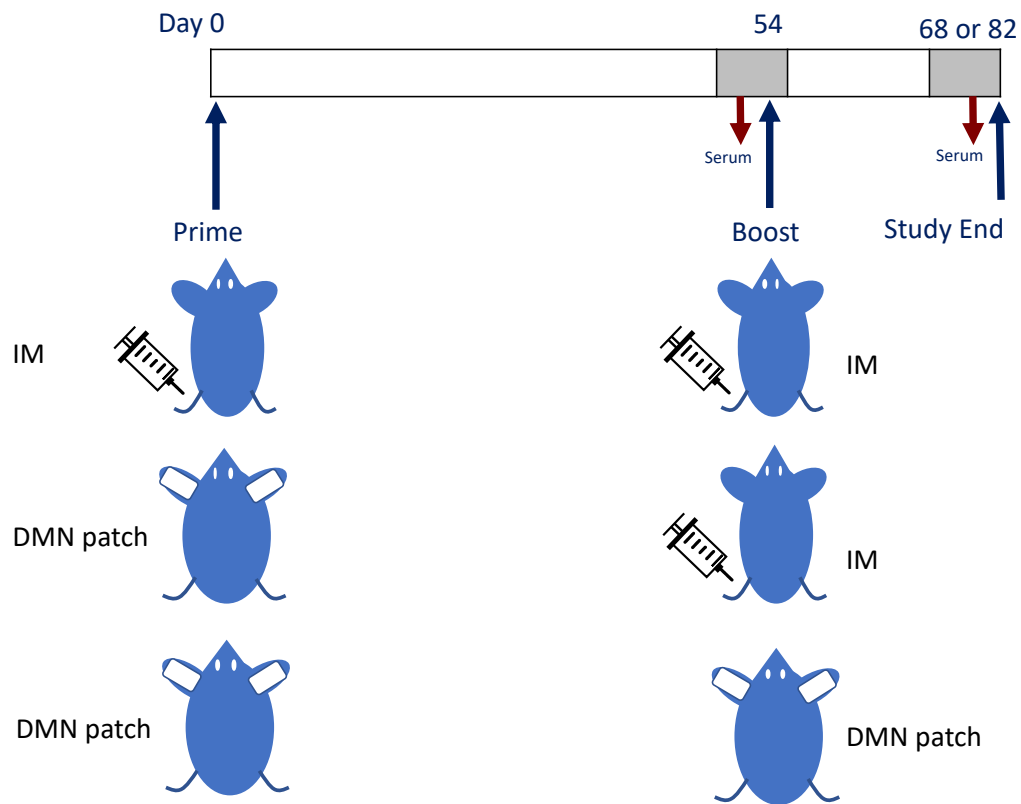

Supplement: Supplementary file 1 [file vaccines-09-00299-s001.pdf]
